# Trivial State Fuzzy Processing for Error Reduction in Healthcare Big Data Analysis towards Precision Diagnosis

**DOI:** 10.3390/bioengineering11060539

**Published:** 2024-05-24

**Authors:** Mohd Anjum, Hong Min, Zubair Ahmed

**Affiliations:** 1 Department of Computer Engineering, Aligarh Muslim University, Aligarh 202002, India; mohdanjum@zhcet.ac.in; 2 School of Computing, Gachon University, Seongnam 13120, Republic of Korea; 3 Department of Zoology, College of Science, King Saud University, Riyadh 11451, Saudi Arabia

**Keywords:** big data, data grouping, fuzzy process, healthcare

## Abstract

There is a significant public health concern regarding medical diagnosis errors, which are a major cause of mortality. Identifying the root cause of these errors is challenging, and even if one is identified, implementing an effective treatment to prevent their recurrence is difficult. Optimization-based analysis in healthcare data management is a reliable method for improving diagnostic precision. Analyzing healthcare data requires pre-classification and the identification of precise information for precision-oriented outcomes. This article introduces a Cooperative-Trivial State Fuzzy Processing method for significant data analysis with possible derivatives. Trivial State Fuzzy Processing operates on the principle of fuzzy logic-based processing applied to structured healthcare data, focusing on mitigating errors and uncertainties inherent in the data. The derivatives are aided by identifying and grouping diagnosis-related and irrelevant data. The proposed method mitigates invertible derivative analysis issues in similar data grouping and irrelevance estimation. In the grouping and detection process, recent knowledge of the diagnosis progression is exploited to identify the functional data for analysis. Such analysis improves the impact of trivial diagnosis data compared to a voluminous diagnosis history. The cooperative derivative states under different data irrelevance factors reduce trivial state errors in healthcare big data analysis.

## 1. Introduction

Big data analysis is a process that helps organize a massive amount of data. It also correlates raw data to processed data, minimizing the complexity of the decision-making process [1]. Healthcare applications contain various datasets that require proper analysis processes to enhance the performance range of the systems [2]. Big data analysis in healthcare improves the overall development and feasibility level of healthcare applications. Medical health records maintained in healthcare contain necessary patient details [3], including personal data, health conditions, types of diseases, medications, and the process of diagnosing diseases for patients. An intelligent-enabled big data analysis technique is commonly used in healthcare applications [4]. This technique analyzes structured and unstructured healthcare data in the management system. The analyzed information produces relevant data for further disease detection and diagnosis processes. The analysis technique also increases the accuracy of disease prediction, reducing latency in providing services to patients [5].

Error reduction is a crucial task to perform in the big data analytics process. Big data analytics eliminates unwanted raw data from the database [6]. Error reduction in data analytics is mainly used to improve the data quality required for further data processing. Errors such as noisy data, negative data, and inconsistent data are present in healthcare management systems [7]. Big data analytics produces optimal information for healthcare applications. Natural language processing (NLP)-based big data analysis is used in healthcare to reduce errors in computational processes [8]. The NLP data processing tool analyzes the critical clinical data necessary for diagnosis [9]. The NLP identifies the negative data that causes errors during data processing and analysis. The identified errors are eliminated immediately to reduce unwanted challenges or issues in big data analytics systems. The NLP-based technique improves the overall functional capability level of the analytics process in healthcare applications [10,11].

Fuzzy methods are widely used in various fields to solve the problems presented in systems. They are also employed in big data analytics to enhance the effectiveness of the systems [12]. A fuzzy-optimized data management (FDM) approach is utilized in big data analytics. This approach employs an extraction technique to extract useful information from datasets [13]. The extracted information provides accumulated data to perform tasks for healthcare systems. The FDM approach analyzes the exact relationship between data and produces feasible data for further analysis [14], thereby improving the feasibility and significance of the analytics process. Additionally, a novel big data analytic technique using a fuzzy similarity measure model is employed in healthcare applications [15]. This analytic technique analyzes the potential data required to perform specific tasks in healthcare centers [16] and manages critical information collected from various divisions. The fuzzy-based technique reduces the data analysis inaccuracy ratio, enhancing the significance of healthcare applications [17].

Now, the key objectives and highlights of the research are stated as:

- To design a Cooperative-Trivial State Fuzzy Processing (CTSFP) method for significant data analysis with possible derivatives.
- To apply fuzzy optimization techniques for grouping data based on functional and irrelevant factors.
- To enhance diagnostic progression by employing various fuzzy derivatives to minimize analysis errors.
- Conduct data and metrics analysis to assess the effectiveness and validate the proposed method.

**Hypothesis 1:**  *Big data analytics positively impacts the innovation system of medical diagnosis.*

**Hypothesis 2:**  *There will be a positive correlation between learning objectives and healthcare data analytics abilities and between learning objectives and performance results in data analytics.*

## 2. Related Works

This literature review highlights significant strides in healthcare data analytics and machine learning applications. In [18], the authors developed a specialized data analytics suite for the management of type 2 diabetes. This suite comprised multi-tier classifiers and advanced analytical methods such as exploratory, predictive, and visual analyses to elucidate the complex interplay between patients’ biological markers, enabling more accurate disease classification and streamlined decision-making processes. A sensor-based data analytics (SBDA) model for real-time patient monitoring in connected healthcare systems addresses the growing need for timely emergency detection and improved efficiency in healthcare applications [19]. Big data analytics gathers various biomedical sensor data for disease detection and prediction. The proposed model is commonly used for real-time patient monitoring, providing feasible emergency detection datasets. These advancements underscore the potential of data-driven approaches to revolutionize disease management and enhance patient care outcomes. Similarly, another study [20] crafted a specialized big data analytics technique for facilitating decision-making within healthcare centers. This technique was designed to gather data from structured and unstructured sources, streamlining the complexity of detection processes. Big data management ensures the generation of optimal datasets, which are essential for effective decision-making. Consequently, the developed technique has been shown to improve the accuracy rate in clinical decision-making, thereby enhancing the feasibility of diagnostic services.

In addressing the pressing concern of data privacy in healthcare, Elayan et al. presented a novel privacy-preserving framework, namely deep federated learning [21]. This framework ensures the safety and confidentiality of patient data while maximizing performance and reducing operational costs by leveraging Internet of Things-enabled devices. Furthermore, a hybrid deep learning technique for healthcare data analytics focuses mainly on disease diagnosis [22]. This technique demonstrates promising results in improving diagnostic accuracy and enhancing the efficiency of diagnostic services, highlighting the transformative impact of machine learning in healthcare. In recent years, wearable sensors have emerged as vital technology applications for monitoring users’ physiological signs, offering valuable insights into health trends. This capability to gather and analyze physiological data has significant implications for enhancing healthcare solutions. An UnSynchronized Sensor Data Analytics (USDA) model has been developed [23], addressing the need to effectively manage wearable device data, particularly in time-sensitive healthcare scenarios. By classifying data based on timing and occurrence frequency, coupled with a diagnosis module, the USDA model identifies defects and addresses missing sensor data crucial for accurate analyses. Utilizing sophisticated machine learning methods enhances diagnostic accuracy and enables timely healthcare solutions, ultimately improving system efficiency and reducing complications in healthcare performance assessment.

In recent literature, researchers have unveiled a groundbreaking healthcare facility management approach by integrating Building Information Modeling (BIM) with big data analytics. This innovative method, rooted in BIM, is designed to harness the information available within building models, thereby generating optimal data for improved detection and diagnosis within healthcare facilities [24]. By leveraging this BIM-based approach, healthcare systems can be transformed, offering enhanced efficiency and effectiveness in patient care delivery. Moreover, a sophisticated smart health monitoring system, grounded in big data principles, has been developed to advance patient care [25]. The proposed model applies Hybrid Dingo Coyote Optimization (HDCO) for optimal feature selection and utilizes a Deep Ensemble Learning algorithm (DEL). This model (HDCO-DEL) accurately classifies various types and classes of medical data, ensuring precise analysis. Integration with Internet of Medical Things devices enables seamless data collection from wireless sensors, thereby minimizing latency in the classification process. Through these innovations, the proposed model significantly elevates the performance standards of healthcare monitoring systems, promising enhanced efficiency and effectiveness in the delivery of patient care. Similarly, Feng et al. introduced a pioneering approach, the confidential information coverage hole prediction, tailored for collecting healthcare big data [26]. Primarily applied within large-scale hybrid wireless sensor networks, this model monitors essential data for detection. Its core objective is leveraging sensor nodes to forecast prior information, mitigating energy consumption in subsequent processes.

Nowadays, healthcare policies worldwide are increasingly emphasizing the importance of leveraging information instruments and digital technologies to enhance public health and quality of life. Therefore, health policies have evolved to incorporate big data analytics as a key driver of digital social innovation in healthcare. In the study [27], the authors introduced digital social innovation-based big data analytics for healthcare applications. Critical analysis is used here to analyze the necessary features and data from medical information. Digital social innovation is mainly used to enhance society’s overall well-being, increasing the efficiency of digital data in smart cities. The introduced method increases the development range of healthcare centers.

Similarly, Khan et al. [28] introduced a systematic analysis framework for healthcare big data analytics to enhance the accuracy and efficiency of disease diagnosis. The model integrates a feature extraction technique to extract pertinent medical data crucial for disease prediction and detection. Notably, the proposed model demonstrates an expanded effectiveness scope for healthcare centers compared to existing approaches.

The prevalence of diabetes mellitus, a chronic metabolic disorder, remains a significant global health challenge, with a concerning proportion of cases going undiagnosed. Early detection and effective management are pivotal in preventing complications and improving patient outcomes. Many studies have focused on the early detection of diabetes by leveraging various machine learning and deep learning models, including stacking algorithms. Therefore, a diabetes patient classification model utilizing the stacking ensemble method is introduced for local healthcare centers [29]. Employing cross-validation techniques enhances the precision of patient classification. By leveraging medical data sourced from local healthcare centers, the model bolsters the robustness of the detection process, ultimately elevating the accuracy of disease detection. In [30], the authors have presented a novel approach to predicting obstructive sleep apnea visit costs in healthcare settings. The method generates viable data inputs for prediction by leveraging electronic healthcare records and Transformer models. These inputs are derived from short visit histories maintained within healthcare centers. Introducing this method maximizes the precision ratio in obstructive sleep apnea prediction, significantly enhancing the robustness of the predictive systems.

Furthermore, Razzak et al. introduced multimedia big data analytics tailored for healthcare centers to improve outcomes in healthcare applications [31]. This automated data processing framework analyzes patients’ health condition data, commonly employed for decision-making and prediction tasks to mitigate computational costs. The experimental findings indicate that the proposed model enhances the quality of patient care, marking a significant advancement in healthcare analytics.

Data storage and transmission formats used by healthcare information technology systems based on CTSFP might be inconsistent and based on diverse standards. Obstacles to interoperability must be overcome so that different systems can communicate data without difficulties and integrate these systems. Unstructured text data, such as patient reports, social media conversations, clinical notes, and medical literature, may be analyzed, and insights may be extracted using NLP algorithms. NLP techniques make it possible to glean useful information from unstructured text, making it easier to accomplish things like sentiment analysis, entity identification, and document summarization.

## 3. Data Collection

**Figure 1:**
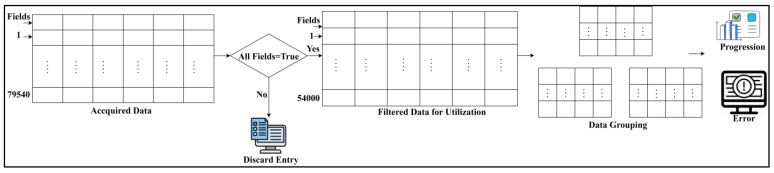
Illustration of healthcare data acquisition and utilization.

The data used in this article is acquired from the “health score” electronic health record for assessment (https://www.kaggle.com/datasets/hansaniuma/patient-health-scores-for-ehr-data, accessed on 15 March 2024). The temperature, pulse, respiratory, blood pressure, dialysis, and imagery information are stored under 79,540 entries. This data is used to classify patient health as severe (or) normal using individual score values. Figure 1 illustrates the acquisition and utilization of healthcare data.

This analysis filters the complete data based on its continuity and field availability. This filtered data is utilized for data grouping and invertible assessments. Of the acquired 79,540 entries, 54,000 are used for this analysis as they possess full values (refer to Figure 1). The progression is analyzed if the detected “severity” is similar to the filtered data entry. The mis-detected (severity as normal (or) vice versa) is regarded as an error in analysis. The CTSFP approach is implemented in real-time scenarios to preprocess the healthcare big data and reduce errors. This process involves noise reduction, outlier detection, data normalization, or fuzzy logic-based processing to enhance data quality and accuracy.

## 4. Cooperative-Trivial State Fuzzy Processing Method

**Figure 2:**
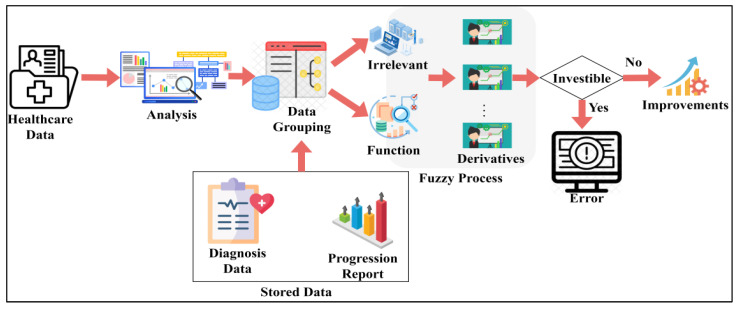
Functional parts of the proposed CTSFP method.

In healthcare management systems, the trivial state reduces big data analytics errors. It defines some significance or uncertainty in the medical data, which decreases the precision of diagnosis. The data points and states are deployed to this trivial state, reducing the importance of the healthcare data. It involves data grouping in this approach where the trivial state data are detected in the healthcare management system and provide efficient results. This concept indicates the big data input and forwards to the detection phase, where the state and data points are recognized. The key idea of this work is to reduce errors and improve the precision of diagnosis. This analysis (CTSFP) is proposed along with this fuzzy model to address the error rate and enhance the diagnosis. In Figure 2, the functional parts of the proposed method are described. Computer resources for the proposed trivial state fuzzy processing include processing power with multi-core processors and adequate memory space for storing healthcare data.

This detection category illustrates the relevant and irrelevant data that are matched with the history of medical data. This analysis is carried out from the stored data, including the diagnosis, which holds the patient’s previous history and the progression report, which illustrates the patient’s update of the prior observation. Based on this stored data, the grouping is performed to handle trivial states better. In this process, a fuzzy processing model is developed to find the n-number of derivations; based on this, invertible and non-veritable are differentiated and the result is provided. The preliminary step in this paper is to handle the trivial state of the healthcare data formulated below.

(1) hn=mg+ot+dp∗Ha+mg+ot+dpgi∑Ha∗Ha+gi∗mg+ot−dp+Ha∑gimg+ot+dp

The trivial state of healthcare data processing, including precision diagnosis and handling, is represented as $h_{n}$, the healthcare data is $H_{a},$ which includes the missing value, outlier, and data points, and they are symbolized as $m_{g},\;o_{t},\;and\;d_{p}$. The diagnosis is described as $g_{i}$. Based on this approach, missing and inconsistent data are detected. This is used to identify the healthcare data better, allowing for better data quality and reliability in this proposed work. This processing step involves the data points where the outliers are detected in this methodology, and from this processing, reduction is observed. In this computation step, trivial data is handled to detect better precision among inconsistent and missing values.

The missing value, outlier, and data points are used to deploy the healthcare data and provide the efficient handling of constraints, which is formulated as mg+ot+dpgi∑Ha. In this category, the analysis examines the trivial state and provides a better progression report for diagnosing the healthcare data. Medical history is analyzed for precision diagnosis, and based on this, the fuzzy model is developed for the n-number of derivations. This trivial state illustrates the derivation from the fuzzy process where the analysis is carried out appropriately. In this method, handling is based on the trivial state of the healthcare data and illustrates the missing value and outlier in the data. From the trivial state handling, the analysis is performed for the varying healthcare data in the stored format, which is equated below.

(2) α=hn+Haot+dp+mg∏ωTs+gi+Ts+Ha∗ω+ct∑mgHa∗gi+Ha+hn−ct+mg+ω(Ha+gi)

The missing and inconsistent data are reasonable in the handling phase, and from this, the analysis is performed, and it is equated as α. A trivial state is represented as $T_{s}$, detection is ω, and inconsistent is described as $c_{t}$. Here, it states that better processing of the diagnosis of healthcare data is needed and provides reliable computation. In this case, the analysis is carried out to improve the data processing. By examining this analysis, the healthcare data are fetched from the database, and from that, matching is achieved with the previous history, and the data is detected similarly to this analysis procedure.

The handling of the trivial state is observed in this approach where the error rate is included, and from this, diagnosis is carried out and is represented as hn+Haot+dp+mg∏ωTs+gi. In this category, the pragmatic data is analyzed for the better output from this trivial state of healthcare data. The existing process detects missing values, and the upcoming methodology is addressed here. In this concept, the trivial state is handled to deploy the quality and reliability of the medical data. Thus, the analysis is carried out for the varying data computation methods in this field, and from this $\omega (H_{a}+g_{i})$ is performed. Then comes the data grouping process in this work, illustrated in the Equation below.

(3) $$\beta =\frac{1}{\omega (p\prime +g_{i})}+\left\{\begin{array}{c} \left(d_{v}+a_{q}\right)*\left\|T_{s}+\alpha \right\|+\left(H_{a}+\frac{a_{q}}{\prod_{h_{n}}T_{s}}\right),\;\forall \;I_{r}\\ \sum_{\alpha }^{d_{v}}\left(a_{q}+d_{n}\right)*\langle m_{g}+T_{s}\rangle +c_{t}-\alpha ,\;\forall \;F_{u} \end{array}\right.$$

The data grouping classification is derived from irrelevant data and functional data, which are symbolized as $I_{r}\;and\;F_{u}$. The data grouping classification is labelled as β, acquiring is $a_{q}$, the progression report is represented as p′, and the n-number of derivations is $d_{n}$. The first stage regards the irrelevant data acquired from the progression report and diagnosis. This history of data and the update of the current scenarios are stored in the database and used for the classification process. The first case indicates the irrelevant process that deploys the acquisition of the data from the dataset and performs a better analysis rate. Handling trivial data involves healthcare data, and it is represented as $\left(H_{a}+\frac{a_{q}}{\prod_{h_{n}}T_{s}}\right)$. The pseudocode for data grouping is presented in Algorithm 1.
**Algorithm 1:** Pseudocode for β
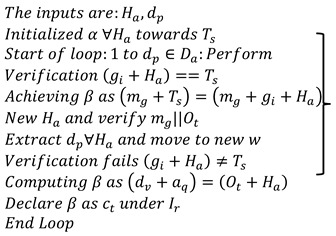

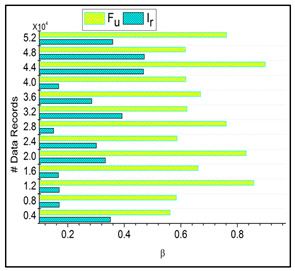


This trivial state indicates healthcare data that includes inconsistent and missing values. The acquisition of the desired data is used to provide trivial data, and the analysis is followed up for the missing value, in which the inconsistency is examined. The second condition is derived from the stored data in which the trivial data are used to better detect the diagnosis among the patients. This approach uses detection to classify the irrelevant and function values. Both classifications are grouped and fetch the data from the storage space. Thus, the data grouping is classified in this concept, and from this, the stored data is observed in the Equation below.

(4) ρ=∑βHagidn+α∗Ts+hn∗gi+p′+aq∗∑Ha+aqTs

This is used to acquire specific data from storage derived from the data grouping. The n-number of derivations is used to perform the better role in this healthcare data processing step, which is described as ρ. This approach includes the classification model for reliable computation of trivial data. Acquiring this data indicates the patient’s previous history, where the matching is processed with the current scenario. In this case, the computation rate is improved by deploying the progression report and diagnosis. This progression report analyses the regular updates for the upcoming process. In Table 1, the input data state is classified and represented.

Table 1 represents the β or α state analysis of the healthcare data accumulated. The F1 to F6 indicates the fields used in the filtered data; their range values are regarded in red (or) green or yellow. Green color denotes the health state low, normal and available ranges from [0,1,2]. Red color indicates the health state high, abnormal and unavailable ranges from [3,4,5]. Considerably, the F6 determines the overall output of the state as α or β. If the abnormal case is high, then α is yet to be completed, for which further derivations are required. In the alternating case, if $c_{t}$ is the maximum possibility, then the data is $T_{s}$ to be denied. Therefore, the state of the data is functional/irrelevant for grouping. Here, the diagnosis and the progressive report indicate a better computation factor and provide efficient derivation matching with the processing history. The n-number of derivations is associated with the analysis where the diagnosis and progressive report are included for further processing, and it is formulated as ∑βHagidn+α. Thus, stored data are used in this trivial processing state where the error is reduced. Since the referred stored data are used in this case, and from this step, the irrelevant and function are differentiated and mapped using the fuzzy optimization model, which is deliberated in the section below.

## 5. Fuzzy Optimization for $F_{u}$ and $I_{r}$ Derivatives

The fuzzy optimization method finds reliable results in the healthcare system where the irrelevant and function are differentiated. This fuzzy logic states whether it is 0 or 1; this paper describes whether it is invertible or non-invertible. This process uses the decision-making method, leading to error identification or improvement. Based on this decision-making approach, the required data are acquired from the stored data, providing a better result. Here, it deploys the trivial state of computation where it indicates the healthcare data for the data grouping methodology. From this, the fuzzification is performed in Equation (5):

(5) $$u_{c}=\left[\left(H_{a}+a_{q}\right)*w_{d}\right]+d_{n}*\sum_{\alpha }\left(\rho +\beta \right)$$

The fuzzification is performed where the input is fetched from the previous step and forwards to the n-number of derivations. The fuzzification is described as $u_{c}$, the forwarding is $w_{d}$. This category uses the analysis to forward the necessary process and provide a reliable classification. This classification states the progressive report and the diagnosis of the healthcare data. Based on this section, the n-number of derivations provides efficient processing in trivial states. For the n-number of derivations, the fuzzification is pragmatic and from which the forwarding is examined in five stages, e.g., $H_{a}+a_{q}$ is large positive, medium positive, small, medium negative, and significant negative.

All these are split in this fuzzification methodology, where healthcare data are acquired for reliable computation from the existing step, including the data grouping. The data grouping indicates the diagnosis and the progressive report, which deploys invertible and non-invertible data. Based on this detection, the performance is measured to process reliable results among the derivation values. The derivation is used to provide better processing, which uses the fuzzy model in this Equation. This fuzzification output equates to the defuzzification in Equation (6).

(6) dz=uc−Ha+dn∗∑aqgi+p′hnct+Ts+ω

**Figure 3:**
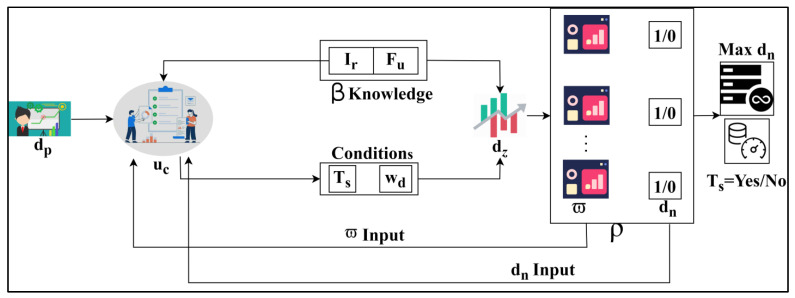
Fuzzification and defuzzification for β.

The defuzzification model is the reverse of the process from the fuzzification where the crisp value is obtained. Equation (6) relies on the healthcare data acquisition and derives the stored data as a diagnosis and progressive report, and the defuzzification is labelled as $d_{z}$. The fuzzification and defuzzification for the grouping are illustrated in Figure 3.

The $u_{c}$ process is initiated with $d_{p}$ input for F1 to F6 entries. This process is later defuzzified through $\left(T_{s},w_{d}\right)$ conditions: $\left(H_{a}+a_{q}\right)==T_{s}$ (or) $\left(g_{i}+H_{a}\right)\neq T_{s}$. The satisfying conditions generate ω and $d_{n}$ which are the defuzzified derivatives used to analyze data states. From these ρ sequences, $\max d_{n}$ and $T_{s}=yes/no$ is obtained. The ρ outputs are further used for sequence classification from $u_{c}$ to verify β knowledge satisfaction (Figure 3). The inconsistent data are acquired from this category and forward the derivation to the next level that indicates the handling phase, and it is formulated as ∑aqgi+p′hnct. Detecting this trivial state is associated with fuzzification, which computes the error reduction. The membership function is accomplished by formulating Equation (7) from above fuzzification and defuzzification.

(7) $$\mu =\{\left(H_{a},\mu \left(m\right)\left(H_{a}\right)\right)|H_{a}\in \beta \}$$

The membership function in fuzzy logic is used to find the 0 or 1 here, and it is defined as either an error or not. If it is not an error, then the improvement is performed. The membership function is m, and the value range from [0, 1] is invertible data and is represented as μ. Universal information is healthcare data; the set of ordered pairs refers to the grouped classifications from the stored data. This observation is performed in the membership function to find the ordered pairs in the trivial state. Here, the invertible data are acquired from the derivation, where the analysis is carried out appropriately. Posted to this membership function in the fuzzy model, the separation is examined from the derivation, whether invertible, non-invertible, or formulated in Equation (8).

(8) $$\vartheta =\left.\begin{array}{c} \left(g_{i}+p^{\prime }\right)*\left(\frac{\alpha \;+\;d_{n}}{\left(H_{a}\right)F_{u}}\right)+T_{s}=0\\ \sum_{T_{s}}\left(I_{r}+d_{n}\right)*\left(g_{i}+p^{\prime }\right)+\beta \neq 0 \end{array}\right\}$$

The separation of trivial states depends on the data that is invertible and non-invertible. Based on this process, the stored data are acquired and perform better data grouping for the irrelevant and function data. The separation is described as ϑ, where the value equal to 0 states the invertible, whereas the value not equal to 0 defines non-invertible. The separation process pseudocode is presented in Algorithm 2.
**Algorithm 2:** Pseudocode for ϑ
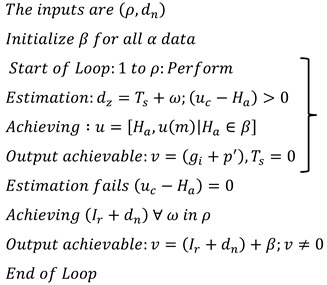

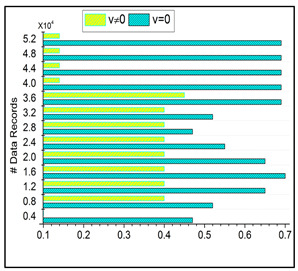


Based on this classification, trivial data are obtained. In this category, detection is observed on the irrelevant function from which the stored data are acquired. Here, data grouping classification is performed for the varying trivial state that deploys the big data as the input for this processing. This derivation is pragmatic for separating invertible and non-invertible data computation. This separation process evaluates the decision-making to find whether it is invertible, equated below.

(9) $$\delta =\left\{\begin{array}{c} 1,\;if\;\prod_{\beta }\left(\alpha +H_{a}\right)*\left(\frac{\mu \;-\;\mu \prime }{g_{i}\;+\;T_{s}}\right)+\vartheta *m\\ 0,\;\;otherwise \end{array}\right.$$

**Figure 4:**
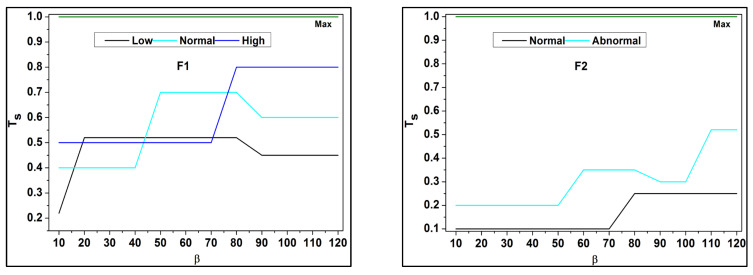

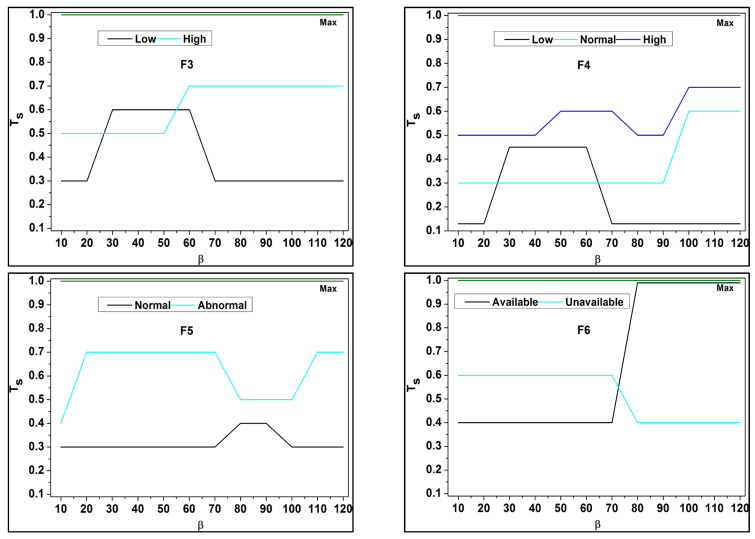
$T_{s}$ Analysis for F1 to F6.

The separation of invertible and non-invertible data follows up the decision-making and provides the changes that occur during the trivial state dispensation, where non-invertible is represented as μ′. The decision-making is δ, defined in *if* and *otherwise* conditions where it deploys the trivial state for the pragmatic healthcare data in this methodology. These functional data are associated with the analysis where the derivations are used to provide better separation among the errors that occur due to the uncertainty due to the missing value. Based on the separation, the fuzzy outputs for $T_{s}$ are analyzed for F1 to F6 in Figure 4.

In Figure 4 above, the classifications under β for different data input fields are validated. The *Y*-axis denotes the trivial state $T_{s}$ and the *X*-axis indicates the data grouping process β. Based on the available $d_{z}$ and it is corresponding $u_{c}$, the $H_{a}$ the analysis is presented. If the $u_{c}$ and $d_{z}$ processes are tallied, then $T_{s}$ is high, otherwise it is low. This demands data acquisition for further ω and $g_{i}$ processes (Figure 4). These derivations are used to examine the invertible, where it is associated with it, and the condition of the *otherwise* function. This condition is followed through the decision-making process, in which fuzzy optimization plays a significant role in this derivation. Here, the invertible condition is satisfied after this identification of functional data runs through the analysis and is equated below.

(10) $$\varphi =\langle \left[\left(\vartheta +\mu \right)*\left(I_{r}-F_{u}\right)\right]+\delta \rangle +u^{\prime }\left(H_{a}\right)+d_{n}$$

The identification of functional data is derived and is formulated as φ; in this stage, the decision-making is carried out for the reliable computation based on the invertible data, and the uncertainty is labelled as u′. The decisions are made from the n-number of derivations and followed up. The healthcare data are integrated with the fuzzy processing in which the membership function is introduced for precision diagnosis. The derivations are associated with irrelevant and functional data processing in this stage. Here, extensive data analysis is performed for the trivial state computation and continues towards the data grouping. Thus, the identification of functional data is examined with the decision-making approach. Then the diagnosis and progression from the stored data are identified and updated based on the current user details. This diagnosis and progression are expressed in Equation (11).

(11) $${\varphi (g}_{i},p\prime )=\frac{1}{d_{n}}*\left[\left(u^{\prime }+H_{a}\right)+\frac{T_{s}+\delta }{\mu *w_{d}}\right]$$

Here, the identification runs through the diagnosis and progression report that includes the status of the patient information. These processes are built into the stored data function. The stored data reflects the changes if any update or changes occur during a short time interval. These changes are identified based on the uncertainty occurrences in the healthcare data for the varying derivations. The relationship between φ and ${\varphi (g}_{i},p\prime )$ for invertible analysis is tabulated in Table 2.

In Table 2, the relationship between ψ and $\psi \left(g_{i},P^{\prime }\right)$ based on valued $d_{z}$ is presented. The field-to-field with normal/available conditions represents the highest relationship (i.e., $\psi =\psi \left(g_{i},P^{\prime }\right)$). The rest of the cases are validated based on $d_{n}$ across v=0 and v≠0 separations. These two cases are derived from multiple $u_{c}$ and $d_{z}$ derivatives such that the u′ is mitigated. Thus, the separation for $\psi =\psi \left(g_{i},p^{\prime }\right)$ incurring instances (above) are high compared to u′ incurring cases (below). This process is repeated until the least possible derivatives u′ are extracted/identified. The n derivations are associated with the trivial state in which the decision-making concept is used for the state forwarding. Based on the update, the changes in the derivation are observed, and the trivial state is used to define the processing step for the different states of the approach. Thus, the identification is carried out for the diagnosis and the progressive report, and the invertible data is detected from the decision-making approach and is equated in the Equation below.

(12) $$\mu \left(\alpha \right)=\left(d_{n}+w_{d}\right)*\prod \left[\left(o_{a}+u\prime \right)+\left(\beta *\delta \right)\right]$$

The invertible data analysis is pragmatic based on the diagnosis and progressive report update. In this approach, functional data is identified, and it is labelled as $o_{a}$; in this classification, it is followed up for the decision-making concept from the fuzzy model. This analysis is executed for the data grouping model and the decision-making of data, whether it is invertible or not. This Equation is used to acquire the input from the big data where the function changes to irrelevant, and then it is said to be invertible; on the other hand, if irrelevant changes to function are observed, it is non-invertible. Thus, the analysis is processed for the invertible data in a trivial state, and the error is detected from this data computation which is equated below.

(13) $$\omega =\left[\frac{\left(T_{s}+F_{u}\right)*\left(h_{n}+o_{a}\right)}{\sum_{\vartheta }^{\alpha }\left(\mu +H_{a}\right)}+\left(\varphi *m_{v}\right)\right]-e_{r}$$

**Figure 5:**
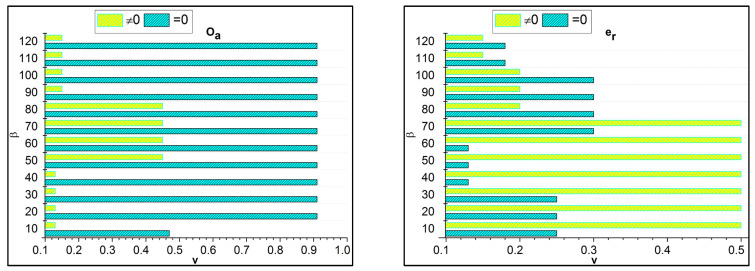
v analysis for $O_{a}$ and $e_{r}$.

The trivial state error is detected from Equation (13), where the functional data are handled for functional usage. The detection is formulated as ω, the error is symbolized as $e_{r}$, and improvement is described as $m_{v}$, where the diagnosis is executed from the derivation from the fuzzy process. In this, the membership function is used to provide the reduction phase for the derivation and order of the pairs. Thus, the error rate is detected in the first derivative and addressed to the upcoming derivations. Based on this error detection in the trivial state, precision diagnosis is improved using this method. With the v for $O_{a}$ and $e_{r}$ using ρ under data grouping towards progression, an error is analyzed, as shown in Figure 5.

In the above assessment for $O_{a}$ and $e_{r}$ under v=0 and v≠0, the β impacts the performance using ρ. The derivatives are abrupt through the max $d_{n}$ and $T_{s}=yes$ conditions for ω improvements. Under different β knowledge conditions, the v separation is performed such that $u_{c}=d_{z}\;\forall \rho$ is satisfied. Hence, the case of β for v=0 is high $\left(O_{a}\right)$ and $low\;\left(e_{r}\right)$ under different data inputs. The filtered data thus is utilized for its intense field-to-field matching for different β (refer to Figure 5). The possible biases caused by fuzzy processing in healthcare data include sampling, measurement, patient selection, and algorithmic biases.

## 6. Performance Assessment

The performance assessment uses analysis rate, data grouping, irrelevance estimation, error, and analysis time. The number of data records considered is 52K, filtered from the dataset inputs, and a maximum of 120 groups are formed. In this assessment, the existing USDA [23], SBDA [19], and HDCO-DEL [25] methods are added along with the proposed CTSFP method for efficacy verification. The data used in this article were acquired from the “health score” electronic health record for assessment (https://www.kaggle.com/datasets/hansaniuma/patient-health-scores-for-ehr-data, accessed on 15 March 2024). The temperature, pulse, respiratory, blood pressure, dialysis, and imagery information are stored under 79,540 entries. This data is used to classify patient health as severe or normal using individual score values.

In healthcare data analysis, the CTSFP method outperforms existing methods like USDA [23], SBDA [19], and HDCO-DEL [25] in terms of error reduction capabilities. Improved diagnosis accuracy and reliability are the results of the proposed method’s use of fuzzy logic-assisted processing to reduce the impact of uncertainties and mistakes in the structure of healthcare data.

By surpassing previous methods in detecting and classifying data important to diagnosis and unrelated data, the technique demonstrates notable advancements in data grouping and irrelevant estimation. The suggested approach improves data classification accuracy and relevance identification by relying on a recent understanding of diagnosis progression and using fuzzy logic in decision-making processes. CTSFP outperforms state-of-the-art approaches regarding concerns about managing healthcare data with trivial states. Compared to more traditional methods, this method can identify and fix data issues, including missing values, inaccurate information, and errors, making it a better foundation for accurate diagnosis and evaluation.

### 6.1. Analysis Rate

**Figure 6:**
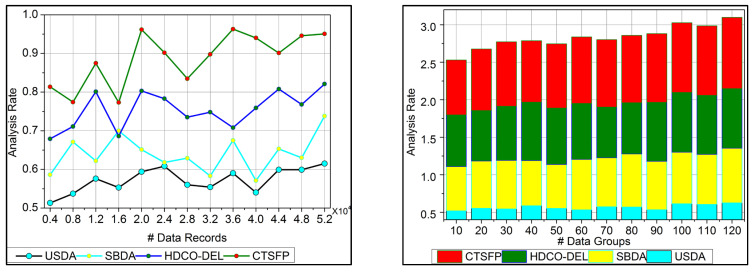
Analysis rate.

The analysis rate for the proposed work increases for the varying trivial state that deploys the fuzzy processing to detect the healthcare data. This approach is based on functional data, which provides the n-number of derivations. Here, the invertible concept is used to find the improvement in the detection process. The big data input is fetched from the healthcare system, and the diagnosis is processed precisely. In this stage, the analysis rate is enhanced by detecting the invertible in the data, which is proposed by the fuzzy optimization method. In this work, data storage is developed for the diagnosis that includes the history of patient data and the progression reports that hold the update of the patient’s health. The trivial state handling of healthcare data addresses the error or the outlier in the input data. This trivial state includes missing or inconsistent datapoints, and it is discussed in Equation (1) and is represented as $\left(\frac{H_{a}}{\sum_{g_{i}}\left(m_{g}+o_{t}+d_{p}\right)}\right)$. The execution of the analysis rate in this work is improved by processing this fuzzy processing under healthcare data (Figure 6).

### 6.2. Data Grouping

**Figure 7:**
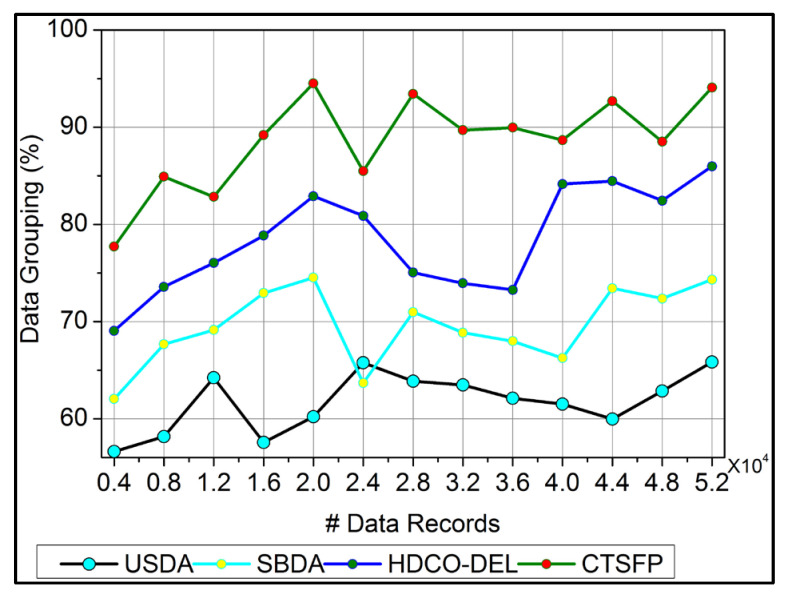
Data grouping.

In Figure 7, data grouping is improved by the classification phase equated in Equation (3). Here, this computation relates to the n-derivation in the fuzzy process and determines the invertible under the input data. In this case, data forwarding to the end process relies on the precision of diagnosis. This concept is proposed to provide irrelevant and functional data in the healthcare system. The evaluation step includes the invertible and non-invertible data used to find the n-number of derivations and provides a better precision diagnosis. The detection process is used to improve the recommendation for the data grouping for healthcare data. The invertible is input to the improvement if the fuzzy process runs accurately for the big data analysis, and it is formulated as $\left(\alpha +H_{a}\right)*\left(\frac{\mu -\mu \prime }{g_{i}+T_{s}}\right)$. Here, the invertible and non-invertible data are examined to find the trivial state. This data grouping is used to represent the diagnosis and the progression report from the invertible data processing. This data grouping is extracted from the stored data, which shows better improvement.

### 6.3. Irrelevance Estimation

**Figure 8:**
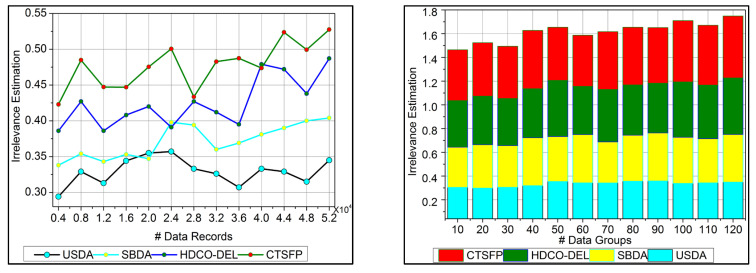
Irrelevance estimation.

The irrelevant estimation shows better improvement based on the trivial state data computation. Here, the decision-making approach, followed by fuzzy logic, is deployed. This fuzzy logic illustrates the membership function that defines the invertible and the data grouping. The irrelevant estimation is identified in this category based on these two structures. This is one approach; the precision diagnosis is used to define the better-stored data and find the trivial state by examining the missing value. The inconsistent and missing values are detected from the trivial state, providing better identification. The diagnosis and progressive report are based on the trivial state handling under healthcare data management, and it is represented as $\langle \left[\left(\vartheta +\mu \right)*\left(I_{r}-F_{u}\right)\right]+\delta \rangle$. The classification phase is used in this work for the functional data and irrelevant identification from the data grouping. From this case, the irrelevant estimation shows the higher value range extracted from the data grouping concept. This estimation phase relies on detecting trivial states for the healthcare data (Figure 8).

### 6.4. Error

**Figure 9:**
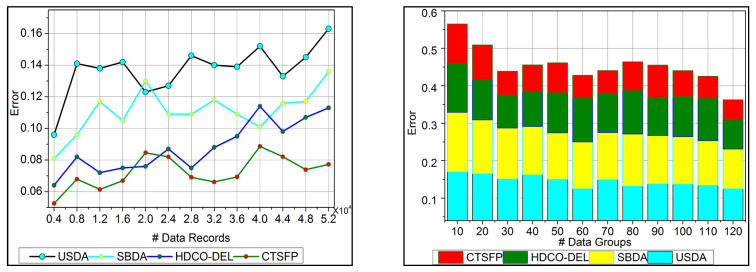
Error.

In Figure 9, the error is detected, and a lesser range is shown for the trivial state by deploying recommendations under the healthcare data. This analysis defines the irrelevant data extraction from the healthcare data and provides the invertible process. This approach is observed for the n-derivation, where the trivial state is examined to find the missing and the inconsistent data from the trivial state handling. This error detection is used to illustrate the data point and the classification under the reduction of constraint for the healthcare data. The detection is followed up for uncertainty and improves the invertible data computation in this approach. The n-derivation is associated with invertible data processing, where the trivial state is used to deploy the fuzzy model. Fuzzification and defuzzification are better used to reference the fuzzy model’s trivial state. Equation (13) is used to find the trivial state of detection and reduce the error factor, and it is equated as $\frac{\left(T_{s}+F_{u}\right)*\left(h_{n}+o_{a}\right)}{\sum_{\vartheta }^{\alpha }\left(\mu +H_{a}\right)}$. Here, the trivial state and functional data are acquired, and the invertible is found, reducing the error.

### 6.5. Analysis Time

**Figure 10:**
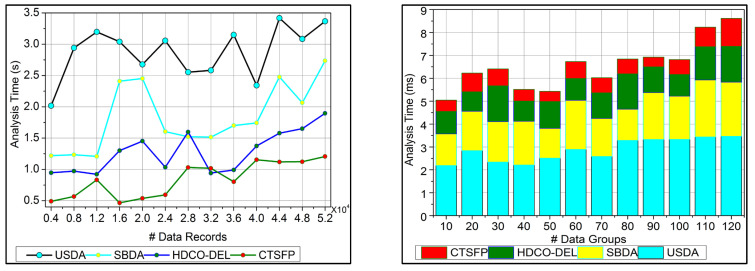
Analysis time.

In Figure 10, the analysis time for the proposed work is reduced based on the data grouping concept. This approach indicates the n-derivation forwarding and provides reliable fuzzy processing. The diagnosis precision is improved in this work by reducing the error in this healthcare data. The uncertainty means the healthcare big data analysis where the identification provides functional data under fuzzy processing. This fuzzy processing is used to provide the invertible under the functional data. This stored data is used to define the diagnosis and the progression report to acquire the data grouping. The derivation is computed by classifying irrelevant or functional data in this optimization process. This identification is used to develop a precision diagnosis with reduced errors. From this stage, the analysis is used to propose the trivial state handling under the irrelevant computation. The analysis time is calculated for the healthcare data processing, and it is reduced and represented as $\left[\left(g_{i}+H_{a}\right)+h_{n}\right]-\left(c_{t}+m_{g}\right)$.

This study utilizes *p*-values to determine whether the sample estimate differs considerably from a hypothesized value. If there was no actual impact, the *p*-value indicates the probability that the observed effect within the research occurred by chance. Statistical significance is traditionally conferred upon data with a *p*-value of <0.05 or <0.01. Within a specified confidence level (e.g., 95%), a confidence interval gives a range of values, one of which is the precise value of the statistical constraint within the specified population. A confidence interval is a range that includes the most likely lower and upper bounds of a connection or difference for a given population. Confidence intervals, as opposed to p-values, provide greater evidence about the accuracy of an estimate; for example, a 95% confidence interval would mean that the range would include the real value in 95% of cases.

In this performance evaluation, the recommended CTSFP is selected for comparison assessment with the existing state-of-the-art methods like USDA, SBDA, and HDCO-DEL methodologies because of their significant advantages to healthcare big data analytics. Important vital aspects covered by each approach include managing data from wearable devices, monitoring patients in real-time, and using an advanced selection of features for accurate evaluation. Essential insights for healthcare management decision-making can be derived from analyzing their performance concerning accuracy, analysis rate, data grouping efficiency, irrelevance estimation, error detection and appropriateness of analysis time for healthcare applications.

In this comparison evaluation, the results across the considered metrics reveal the advantages and disadvantages of each strategy, giving helpful information about the best method to put them into practice in analyzing healthcare big data. For efficient and precise processing of missing information from sensors, the USDA model performs exceptionally well in the real-time monitoring of patients. Disease detection and forecasting are two areas where the SBDA model shines, demonstrating its strength in times of crisis. In contrast, the HDCO-DEL model is scalable and displays remarkable accuracy in medical data classification, making healthcare surveillance techniques more dependable.

Despite the methods’ strengths, they all have drawbacks, such as computational inefficiency and problems with data analysis. In contrast, the suggested CTSFP approach is used to preprocess healthcare big data to increase accuracy, data analysis time, and resilience to overcome these restrictions. The CTSFP method is an innovative new direction for healthcare management practices since it uses data normalization, noise reduction, and outlier detection to enhance healthcare analytics. Additional empirical testing is required to determine the method’s practical usefulness. Still, it offers a fresh perspective on the challenges associated with big data healthcare analysis and could lead to more reliable decision-making in the healthcare domain.

## 7. Conclusions

This article introduces the CTSFP method to improve the efficacy of healthcare data analytics. The proposed method separates healthcare data analysis toward invertible improvement and error reduction. This method utilizes fuzzy optimization to identify irrelevant and functional data based on real-time measures. The fuzzy derivatives satisfying invertible conditions are utilized under error reduction and diagnosis-oriented improvements. Based on the separation, data grouping for irrelevant and functional input is validated under fuzzification and defuzzification processes that extract data deviations separately. Therefore, the functional data for diagnosis improvements are augmented for further irrelevant data reduction. Thus, the proposed method is introduced for significant data analysis with the possibility of derivatives detected using the fuzzy optimization method. Since the trivial state error rate is reduced, healthcare big data analysis is enhanced. Data storage and transmission formats used by healthcare information technology systems might be inconsistent and based on diverse standards. The proposed scheme has overcome obstacles to interoperability so that different systems can communicate data without any problems, integrating these systems.

## Figures and Tables

**Table 1:** Input data state classification.

	F1	F2	F3	F4	F5	F6	State
	Low/Normal/ High	Normal/ Abnormal	Low/High	Low/Normal/ High	Normal/ Abnormal	Available/ Unavailable	
$d_{p}$	●● ●	● ●	●●	●●●	●●	●	β
$I_{r}$	● ●●	● ●	●●	● ● ●	● ●	●	α
$F_{u}$	●● ●	● ●	● ●	● ●●	●●	●	β
$c_{t}$	●● ●	● ●	● ●	● ●●	● ●	●	α

**Table 2:** Invertible analysis using φ and ${\varphi (g}_{i},p\prime )$.

φ	F1	F2	F3	F4	F5	F6	${\varphi (g}_{i},p\prime )$
	Low/Normal/ High	Normal/ Abnormal	Low/ High	Low/Normal/ High	Normal/ Abnormal	Available/ Unavailable	
F1	0.65	0.52	0.58	0.65	0.71	0.82	Low
	1	0.61	0.65	0.74	0.75	0.85	Normal
	0.18	0.68	0.82	0.87	0.65	0.93	High
F2	0.25	1	0.91	0.91	0.74	0.87	Normal
	0.36	0.15	0.86	0.85	0.72	0.87	Abnormal
F3	0.25	0.17	1	0.96	0.81	0.95	Low
	0.41	0.21	0.25	0.87	0.92	0.97	High
F4	0.39	0.35	0.31	0.92	0.98	0.97	Low
	0.42	0.36	0.42	1	0.97	0.95	Normal
	0.39	0.41	0.52	0.41	0.84	0.97	High
F5	0.42	0.5	0.42	0.48	1	0.99	Normal
	0.45	0.36	0.11	0.32	0.5	0.87	Abnormal
F6	0.48	0.29	0.28	0.36	0.48	1	Available
	0.39	0.31	0.32	0.24	0.42	0.49	Unavailable

## Data Availability

The data used in this study is available from Kaggle at the following link: https://www.kaggle.com/datasets/hansaniuma/patient-health-scores-for-ehr-data.

## References

[1] Yan L., Huang W., Wang L., Feng S., Peng Y., Peng J. (2019). Data-enabled digestive medicine: A new big data analytics platform. IEEE/ACM Trans. Comput. Biol. Bioinform..

[2] Biswas R. (2022). Outlining big data analytics in health sector with special reference to COVID-19. Wirel. Pers. Commun..

[3] Batko K., Ślęzak A. (2022). The use of Big Data Analytics in healthcare. J. Big Data.

[4] Hassan S., Dhali M., Zaman F., Tanveer M. (2021). Big data and predictive analytics in healthcare in Bangladesh: Regulatory challenges. Heliyon.

[5] Chao K., Sarker M.N.I., Ali I., Firdaus R.R., Azman A., Shaed M.M. (2023). Big data-driven public health policy making: Potential for the healthcare industry. Heliyon.

[6] Koeck J.A., Young N.J., Kontny U., Orlikowsky T., Bassler D., Eisert A. (2021). Interventions to reduce pediatric prescribing errors in professional healthcare settings: A systematic review of the last decade. Pediatr. Drugs.

[7] Ahsani-Estahbanati E., Sergeevich Gordeev V., Doshmangir L. (2022). Interventions to reduce the incidence of medical error and its financial burden in health care systems: A systematic review of systematic reviews. Front. Med..

[8] Gates P.J., Hardie R.A., Raban M.Z., Li L., Westbrook J.I. (2021). How effective are electronic medication systems in reducing medication error rates and associated harm among hospital inpatients? A systematic review and meta-analysis. J. Am. Med. Inform. Assoc..

[9] Schiavone F., Sabetta A., Leone D., Chiao B. (2021). Industrial convergence and industrial crisis: A situational analysis about precision medicine during the Covid-19 pandemic. IEEE Trans. Eng. Manag..

[10] Fioretos T., Wirta V., Cavelier L., Berglund E., Friedman M., Akhras M., Botling J., Ehrencrona H., Engstrand L., Helenius G. (2022). Implementing precision medicine in a regionally organized healthcare system in Sweden. Nat. Med..

[11] Geissler J., Makaroff L.E., Söhlke B., Bokemeyer C. (2023). Precision oncology medicines and the need for real world evidence acceptance in health technology assessment: Importance of patient involvement in sustainable healthcare. Eur. J. Cancer.

[12] Yan F., Huang H., Pedrycz W., Hirota K. (2024). A disease diagnosis system for smart healthcare based on fuzzy clustering and battle royale optimization. Appl. Soft Comput..

[13] Ghorbani A., Davoodi F., Zamanifar K. (2023). Using type-2 fuzzy ontology to improve semantic interoperability for healthcare and diagnosis of depression. Artif. Intell. Med..

[14] Ahmed A., Xi R., Hou M., Shah S.A., Hameed S. (2023). Harnessing big data analytics for healthcare: A comprehensive review of frameworks, implications, applications, and impacts. IEEE Access.

[15] Rehman A., Naz S., Razzak I. (2022). Leveraging big data analytics in healthcare enhancement: Trends, challenges and opportunities. Multimed. Syst..

[16] Taipalus T., Isomöttönen V., Erkkilä H., Äyrämö S. (2022). Data Analytics in Healthcare: A Tertiary Study. SN Comput. Sci..

[17] Badawy M., Ramadan N., Hefny H.A. (2023). Healthcare predictive analytics using machine learning and deep learning techniques: A survey. J. Electr. Syst. Inf. Technol..

[18] Philip N.Y., Razaak M., Chang J., O’Kane M., Pierscionek B.K. (2022). A data analytics suite for exploratory predictive, and visual analysis of type 2 diabetes. IEEE Accesss.

[19] Harb H., Mansour A., Nasser A., Cruz E.M., de la Torre Diez I. (2020). A sensor-based data analytics for patient monitoring in connected healthcare applications. IEEE Sens. J..

[20] Hussain F., Nauman M., Alghuried A., Alhudhaif A., Akhtar N. (2023). Leveraging Big Data Analytics for Enhanced Clinical Decision-Making in Healthcare. IEEE Access.

[21] Elayan H., Aloqaily M., Guizani M. (2021). Sustainability of healthcare data analysis IoT-based systems using deep federated learning. IEEE Internet Things J..

[22] Shafqat S., Fayyaz M., Khattak H.A., Bilal M., Khan S., Ishtiaq O., Abbasi A., Shafqat F., Alnumay W.S., Chatterjee P. (2021). Leveraging deep learning for designing healthcare analytics heuristic for diagnostics. Neural Process. Lett..

[23] Alfarraj O., Tolba A. (2021). Unsynchronized wearable sensor data analytics model for improving the performance of smart healthcare systems. J. Ambient. Intell. Humaniz. Comput..

[24] Demirdöğen G., Işık Z., Arayici Y. (2023). BIM-based big data analytic system for healthcare facility management. J. Build. Eng..

[25] Abidi M.H., Umer U., Mian S.H., Al-Ahmari A. (2023). Big Data-based Smart Health Monitoring System: Using Deep Ensemble Learning. IEEE Access.

[26] Feng J., Chen H., Deng X., Yang L.T., Tan F. (2020). Confident information coverage hole prediction and repairing for healthcare big data collection in large-scale hybrid wireless sensor networks. IEEE Internet Things J..

[27] Batko K. (2023). Digital social innovation based on Big Data Analytics for health and well-being of society. J. Big Data.

[28] Khan S., Khan H.U., Nazir S. (2022). Systematic analysis of healthcare big data analytics for efficient care and disease diagnosing. Sci. Rep..

[29] Reza M.S., Amin R., Yasmin R., Kulsum W., Ruhi S. (2024). Improving diabetes disease patients’ classification using stacking ensemble method with PIMA and local healthcare data. Heliyon.

[30] Chen Z., Siltala-Li L., Lassila M., Malo P., Vilkkumaa E., Saaresranta T., Virkki A.V. (2023). Predicting Visit Cost of Obstructive Sleep Apnea using Electronic Healthcare Records with Transformer. IEEE J. Transl. Eng. Health Med..

[31] Razzak I., Eklund P., Xu G. (2022). Improving healthcare outcomes using multimedia big data analytics. Neural Comput. Appl..

